# Prevalence and risk factors for initiating tobacco and alcohol consumption in adolescents living in urban and rural Ethiopia

**DOI:** 10.1016/j.puhe.2019.05.029

**Published:** 2019-09

**Authors:** S. Getachew, S. Lewis, J. Britton, W. Deressa, A.W. Fogarty

**Affiliations:** a Department of Preventive Medicine, School of Public Health, College of Health Sciences, Addis Ababa University, Addis Ababa, Ethiopia; b Division of Epidemiology and Public Health, University of Nottingham, Nottingham, UK

**Keywords:** Alcohol, Tobacco, adolescents, Ethiopia, football, advertising

## Abstract

**Objectives:**

African countries are potential high growth markets for the alcohol and tobacco industries. This study aimed to identify exposures that are associated with initiating use of alcohol and tobacco products in young people living in Ethiopia. As televised football and Internet viewing are media through which products can be promoted to this population efficiently, these risk factors were of particular interest.

**Study design:**

This is a cross-sectional study.

**Methods:**

Data were collected using a self-administered questionnaire of 3967 children aged 13–19 years in 20 high schools in urban and rural Ethiopia on consumption and risk factors for alcohol and tobacco use in 2016, as well as exposure to potential sources of advertising.

**Results:**

Eight percent of respondents reported having ever smoked and 3% were current smokers. Twenty-nine percent reported ever having used alcohol, and 14% were current users. Risk factors for ever smoking included father smoking (odds ratio [OR] 1.95; 95% confidence interval [CI]: 1.21 to 3.15), mother smoking (OR 3.90; 95% CI: 1.63 to 9.33), best friend smoking (OR 5.86; 95% CI: 4.31 to 7.96) and home Internet access (OR 1.75; 95% CI: 1.35 to 2.27). There was a very strong positive association between ever having smoked cigarettes and ever having tried alcohol (*P* < 0.001). Risk factors for currently drinking alcohol included father drinking (OR 1.45; 95% CI: 1.05 to 2.01), mother drinking (OR 2.00; 95% CI: 1.44 to 2.77), home Internet access (OR 1.53; 95% CI: 1.24 to 1.90) and regular watching of televised football (OR 2.44 compared to those who do not; 95% CI: 1.58 to 3.78).

**Conclusions:**

As in rich countries, tobacco and alcohol use among Ethiopian teenagers increases among those exposed to family and peer use but are also more common among those accessing the Internet and, for alcohol, those watching televised football. The effect of watching televised football on alcohol use, at least, is likely to be due to exposure to advertising.

## Highlights

• Rapid economic development is changing the environment in developing countries rapidly, generating a new potential market for the alcohol and tobacco industries.
• Fourteen percent of adolescents living in Ethiopia are regular consumers of alcohol, and 3% regularly smoke cigarettes.
• Regular watching of international football on television is associated with being a current consumer of alcohol.
• Public health strategies to modify alcohol and tobacco usage in adolescents living in Ethiopia need to consider that the Internet and televised football matches are potential sources of exposure to advertising aiming to promote consumption.

## Introduction

Tobacco smoking is probably the largest long-term challenge to public health in developing countries. Smoking kills more than 7 million people each year,^1^ and around 80% of the world's smokers live in low- or middle-income countries.^2^ Similarly, alcohol is emerging as an increasing public health problem in developing countries, and particularly in young adults.^3^ Ethiopia is an East African country with a population of 104 million individuals, a gross national product of $783 per capita in 2016, and an annual economic growth rate of approximately 10% in recent years.^4^ Ethiopia, therefore, has high potential for development by and represents a valuable future market opportunity for both the alcohol and tobacco industries.

Currently, smoking remains relatively uncommon with an estimated prevalence for adults in 2011 of 4.1% in the country as a whole, but with wide regional variations.^5^ The prevalence of current smoking in adolescents living in Addis Ababa in 2003 was 2.9%, and data from a similar aged population living in eastern Ethiopia in 2010 suggested 4.2% were smoking daily.^6^ However, alcohol consumption among young people is already a substantial problem in Ethiopia; studies among secondary school students report estimates that up to 50% consume alcohol,^7^ and this is also recognised as a broader public health problem among young people living in Eastern Africa.^8^

The factors driving the uptake of tobacco and alcohol consumption among young people in Ethiopia have not been widely investigated. However, one key potential risk factor for alcohol consumption is exposure to alcohol advertising in televised football, which has been shown to be an important advertising vehicle for alcohol in the UK,^9^ and is very popular in Ethiopia. Because exposure to alcohol advertising is associated with subsequent initiation of alcohol consumption in young people,^10^ this is a particularly important risk factor of interest. To help inform effective public health interventions to improve future societal health, this study has therefore measured the prevalence of alcohol and tobacco consumption and estimated effects of putative risk factors including watching football, in young people living in Ethiopia.

## Methods

### Study population

Ten rural and ten urban schools with respective rolls of 8304 and 13,528 were selected from a list of schools identified from the Addis Ababa City Administration, Oromia Regional State and Southern Nations, Nationalities, and People's Region (SNNPR) education offices. The rural schools were in Oromia state (eight) and SNNPR (two), which are located approximately 45–160 km from Addis Ababa, while the urban schools were in Addis Ababa. All schools that were invited to participate agreed and did so. There were 184 urban schools and 52 rural schools in this area, although we targeted schools that were accessible and representative of the area as whole.

A random sample from grades 9–12 was selected using the school register. A total of 5000 questionnaires were allocated equally to the urban and rural schools and then distributed throughout the schools by assigned facilitators and the respective school teachers being allocated proportionately to school and class size. Children with mental or visual impairment or who were absent through illness at the time of data collection were not eligible for the study.

### Data collection

The questionnaire was written in either Amharic or Afaan Oromo (depending on the local language) and piloted in 100 individuals in Addis Ababa before use in the survey. Questionnaire surveys were carried out by data collection supervisors who, between April and June 2016, distributed questionnaires to all children aged 13–19 years who were asked to complete it personally. The questionnaire asked a range of questions including use of alcohol and tobacco products by the respondents and also their access to the Internet and frequency of watching football on television. All responses were self-reported, and it was not possible to collect any objective measures on the exposures of interest.

### Statistical analysis

Data were entered into an SPSS electronic database and cleaned by checking for implausible values. Ever smokers were defined as those who had ever tried smoking, current smokers as those who reported having smoked cigarettes in the last 30 days, ever having drunk alcohol as those who reported ever having drunk beer, wine or any other alcohol product and current drinking as those who reported regularly drinking beer, wine or any other alcohol product in the last 30 days or now.

The statistical analysis was predominantly descriptive, using Chi-squared testing to test for statistical differences between categorical groups and a *P* value of <0.05 to define a significant association. Because age 13–19 years was an inclusion criterion, those with missing age were excluded from the primary analysis, but the effect of including them on smoking and alcohol prevalence estimates was explored in a sensitivity analysis. Variables showing significant association with tobacco or alcohol consumption were entered simultaneously into a multivariate logistic model to test independence of observed associations. Variables that were potentially collinear, for example, age and educational stage, were not included in the same model but entered one at a time to determine the stronger effect. All available data were used, but the proportion of missing data was also considered in determining which variables to enter to a model. Linearity of effects was tested using the likelihood ratio test. A final model was fitted for each outcome including only variables significant after mutual adjustment and allowing for clustering by school using the robust cluster estimator in Stata. Data analysis used Stata statistical software (version 14, Texas USA).

## Results

Of the 5000 young people invited to complete the questionnaire, 4705 (94.1%) did so. The self-reported ages of respondents ranged from 6 to 35 years, with 553 missing values; after excluding those aged younger than 13 years (13 respondents) or older than 19 years (172 respondents) and those with missing data on age, 3967 questionnaires remained for the primary analysis (Table 1).

**Table 1:** Description of the study population.

	Urban [n (%)]	Rural [n (%)]	All
**Total**	2139	1828	3967
Male	942 (44.5)	873 (48.1)	1815 (46.2)
Female	1175 (55.5)	942 (51.9)	2117 (53.8)
Missing	22	13	35
**Age (years)**			
13	5 (0.2)	9 (0.5)	14 (0.3)
14	70 (3.3)	36 (2.0)	106 (2.7)
15	330 (15.4)	270 (14.8)	600 (15.1)
16	430 (20.1)	512 (28.0)	942 (23.8)
17	572 (26.7)	503 (27.5)	1075 (27.1)
18	596 (27.9)	386 (21.1)	982 (24.7)
19	136 (6.4)	112 (6.1)	248 (6.2)
**Grade**			
9	733 (34.4)	808 (44.5)	1541 (39.1)
10	266 (12.5)	490 (27.0)	756 (19.2)
11	712 (33.4)	400 (22.0)	1112 (28.2)
12	418 (19.6)	117 (6.5)	535 (13.6)
Missing	10	13	23
**Ethnicity**			
Oromo	398 (19.2)	1148 (64.1)	1546 (40.1)
Amhara	823 (39.8)	330 (18.4)	1153 (29.9)
Gurage	356 (17.2)	206 (11.5)	562 (14.6)
Tigre	267 (12.9)	45 (2.5)	312 (8.1)
Wolayta	48 (2.3)	10 (0.6)	58 (1.5)
Silite	82 (4.0)	25 (1.4)	107 (2.8)
Other	95 (4.6)	26 (1.5)	121 (3.1)
Missing	70	38	108
**Religion**			
Orthodox Christian	1527 (72.2)	1313 (73.1)	2840 (72.6)
Protestant	165 (7.8)	251 (14.0)	416 (10.6)
Catholic	37 (1.7)	48 (2.7)	85 (2.2)
Muslim	367 (17.4)	156 (8.7)	523 (13.4)
Other	18 (0.8)	28 (1.6)	46 (1.2)
Missing	25	32	57

### Smoking

A total of 323 (8.5%) respondents reported that they had ever smoked (172 missing responses) (Table 2), 97 (2.6%) that they had smoked in the past 30 days and 68 (1.8%) that they had smoked in the last seven days. The median cigarette consumption in the last week for the individuals who gave data for each day was 16 (range 0–70).

**Table 2:** Prevalence and risk factors for ever and current smoking cigarettes.

	Total	Ever smoking [n (%)]	Current smoking^a^ [n (%)]
**Total**	3795	323 (8.5)	97 (2.6)
Urban	2069	200 (9.7%)	78 (3.3)
Rural	1726	123 (7.1%)	51 (2.6)
		*P* = 0.005	*P* = 0.14
**School**			
Government school	2637	215 (8.1)	67 (2.5)
Private school	1158	108 (9.3)	30 (2.6)
		*P* = 0.2	*P* = 0.9
**Age (years)**			
13 or 14	109	12 (11.0)	5 (4.6)
15	568	13 (2.3)	4 (0.7)
16	891	62 (7.0)	13 (1.5)
17	1035	87 (8.4)	23 (2.2)
18	949	104 (11.0)	34 (3.6)
19	243	45 (18.5)	18 (7.4)
		*P* < 0.001	*P* < 0.001
**Gender**			
Male	1747	220 (12.6)	64 (3.7)
Female	2014	96 (4.8)	27 (1.3)
		*P* < 0.001	*P* < 0.001
**Grade**			
9	1449	105 (7.2)	29 (2.0)
10	724	52 (7.2)	19 (2.6)
11	1077	104 (10.0)	31 (2.9)
12	526	62 (11.8)	18 (3.4)
		*P* = 0.003	*P* = 0.3
**Ethnicity**			
Oromo	1447	101 (7.0)	32 (2.2)
Amhara	1122	81 (7.2)	19 (1.7)
Gurage	548	48 (8.8)	9 (1.6)
Tigre	306	40 (13.1)	15 (4.9)
Wolayta	56	11 (19.6)	7 (12.5)
Silite	97	14 (14.4)	4 (4.1)
Other	119	22 (18.5)	11 (9.2)
		*P* < 0.001	*P* < 0.001
**Religion**			
Orthodox Christian	2732	208 (7.6)	56 (2.1)
Protestant	391	26 (6.6)	9 (2.3)
Catholic	80	7 (8.7)	4 (5.0)
Muslim	493	61 (12.4)	15 (3.0)
Other	44	10 (22.7)	7 (15.9)
		*P* < 0.001	*P* < 0.001
**Father smokes?**			
Yes	174	43 (24.7)	19 (10.9)
No	3187	226 (7.1)	59 (1.8)
Not applicable	258	30 (11.6)	9 (3.5)
		*P* < 0.001	*P* < 0.001
**Mother smokes?**			
Yes	44	21 (47.7)	17 (38.6)
No	3343	243 (7.3)	56 (1.7)
Not applicable	220	35 (15.9)	13 (5.9)
		*P* < 0.001	*P* < 0.001
**Best friend smokes?**			
Yes	378	136 (36.0)	71 (18.9)
No	2581	126 (4.9)	14 (0.5)
Do not know	668	44 (6.6)	4 (0.6)
		*P* < 0.001	*P* < 0.001

a Having consumed tobacco products in the past 30 days.

**Table 3:** Prevalence and risk factors for alcohol use.

	Total	Ever drunk alcohol [n (%)]	Current regular alcohol use [n (%)]
**Total**	3899	1116 (28.6)	551 (14.1)
Urban	2097	569 (27.1)	227 (10.8)
Rural	1800	464 (25.8)	324 (18.0)
		*P* = 0.3	*P* < 0.001
**Schools**			
Government school	2739	702 (25.6)	442 (16.1)
Private school	1158	331 (28.6)	109 (9.4)
		*P* = 0.06	*P* < 0.001
**Age (years)**			
13 or 14	117	17 (14.5)	9 (7.7)
15	593	99 (16.7)	46 (7.8)
16	930	212 (22.8)	106 (11.4)
17	1053	291 (27.6)	151 (14.3)
18	964	383 (39.7)	185 (19.2)
19	242	114 (47.1)	54 (22.3)
		*P* < 0.001	*P* < 0.001
**Gender**			
Male	1793	674 (37.6)	338 (18.8)
Female	2072	428 (20.7)	206 (9.9)
		*P* < 0.001	*P* < 0.001
**Grade**			
9	1519	378 (24.9)	203 (13.4)
10	742	212 (28.6)	135 (18.2)
11	1095	328 (29.9)	145 (13.2)
12	521	193 (37.0)	65 (12.5)
		*P* < 0.001	*P* = 0.005
**Ethnicity**			
Oromo	1519	463 (30.5)	268 (17.6)
Amhara	1140	336 (29.5)	157 (13.8)
Gurage	551	103 (18.7)	33 (6.0)
Tigre	310	119 (38.4)	49 (15.8)
Wolayta	56	17 (30.4)	9 (16.1)
Silite	102	18 (17.6)	14 (13.7)
Other	120	38 (31.7)	11 (9.2)
		*P* < 0.001	*P* < 0.001
**Religion**			
Orthodox Christian	2804	926 (33.0)	443 (15.8)
Protestant	405	84 (20.7)	49 (12.1)
Catholic	83	26 (31.3)	21 (25.3)
Muslim	505	48 (9.5)	21 (4.2)
Other	46	12 (26.1)	6 (13.0)
		*P* < 0.001	*P* < 0.001
**Father drinks?**			
Yes	1359	544 (40.0)	256 (18.8)
No	1937	383 (19.8)	184 (9.5)
Not applicable	366	113 (30.9)	66 (18.0)
		*P* < 0.001	*P* < 0.001
**Mother drinks?**			
Yes	511	229 (44.8)	121 (24.0)
No	2908	722 (24.8)	330 (11.3)
Not applicable	270	85 (31.5)	48 (17.8)
		*P* < 0.001	*P* < 0.001
**Friends who drink?**			
<10%	1228	419 (34.1)	198 (16.1)
10–40%	361	172 (47.6)	81 (22.4)
>40%	480	304 (63.3)	181 (37.7)
Missing	1830		
		*P* < 0.001	*P* < 0.001

Of the 323 who had ever smoked, 262 gave their age at starting to smoke cigarettes, with a median age of 15 years. The most common sources of cigarettes were purchase from a supermarket, other shop or street market. Of those who had been into a shop to buy cigarettes, either for themselves or someone else (n = 944), 30.7% said it was very or fairly easy to do so. When asked about using water pipes for smoking, 903 (25.7% of 3509 responses) had heard of them, and 229 (7.1%) had tried them, with 25 (0.8%) using them regularly.

### Risk factors for smoking

In univariate analysis, age, sex, urban/rural location, grade, religion, ethnicity, father and mother smoking and best friend smoking were significant predictors of ever trying smoking (Table 2). The risk of ever smoking was higher in urban schools, among boys, and tended to increase with age. Ever smoking was higher in the Tigre and Wolayta ethnic groups and among Muslims compared to Christians. Ever smoking was more likely in those with parents who smoke, especially a mother who smokes, and in individuals whose best friend smokes. Ever smoking was also more common among those who watched football daily (Table 4), especially those who supported UK teams such as Manchester United, Chelsea or Liverpool. Ever smoking was also more common among those with Internet access at home.

**Table 4:** Sources of exposure to smoking and alcohol promotions.

	Total who provided data on smoking [n (%)]	Ever tried smoking [n (%)]	Total who provided data on alcohol [n (%)]	Ever tried alcohol [n (%)]	Current regular drinking [n (%)]
**Watching football**					
Do not like/never	1284 (35.9)	88 (6.8)	1305 (35.5)	274 (21.0)	127 (9.7)
Once a month	628 (17.6)	37 (5.9)	657 (17.9)	148 (22.5)	65 (9.9)
Weekly	1343 (37.6)	125 (9.3)	1388 (37.7)	474 (34.1)	240 (17.3)
Daily	319 (8.9)	51 (16.0)	329 (8.9)	148 (45.0)	86 (26.1)
		*P* < 0.001		*P* < 0.001	*P* < 0.001
**Favourite football team**					
Manchester United	1338 (45.8)	132 (9.9)	1370 (45.6)	456 (33.3)	219 (16.0)
Chelsea	303 (10.4)	33 (10.9)	309 (10.3)	95 (30.7)	51 (16.5)
Liverpool	99 (3.4)	14 (14.1)	97 (3.2)	33 (34.0)	16 (16.5)
Manchester City	155 (5.3)	6 (3.9)	162 (5.4)	54 (33.3)	33 (20.4)
Arsenal	958 (32.8)	67 (7.0)	994 (33.1)	264 (26.6)	127 (12.8)
Other	66 (2.3)	8 (12.1)	73 (2.4)	24 (32.9)	12 (16.4)
		*P* = 0.006		*P* = 0.019	*P* = 0.10
**Home Internet**					
No	1869 (52.3)	113 (6.0)	1941 (52.7)	464 (23.9)	235 (12.1)
Yes	1702 (47.7)	185 (10.9)	1741 (47.3)	592 (34.0)	285 (16.4)
		*P* < 0.001		*P* < 0.001	*P* < 0.001
**Mobile phone**					
No	730 (20.7)	54 (7.4)	774 (21.3)	148 (19.1)	87 (11.2)
Yes	2793 (79.3)	243 (8.7)	2858 (78.7)	888 (31.1)	421 (14.7)
		*P* = 0.3		*P* < 0.001	*P* = 0.022
**Seen cigarette brand on TV in the last 30 days**					
No	2986 (82.9)	214 (7.2)		–	–
Yes	616 (17.1)	84 (13.6)		–	–
		*P* < 0.001			
**Possess item with cigarette brand logo**					
No	3368 (94.4)	240 (7.1)		–	–
Yes	201 (5.6)	61 (30.3)		–	–
		*P* < 0.001			
**Seen tobacco branding on billboard recently**					
No	3104 (88.1)	230 (7.4)		–	–
Yes	418 (11.9)	69 (16.5)		–	–
		*P* < 0.001			
**Seen tobacco adverts in papers recently**					
No	3108 (90.7)	235 (7.6)		–	–
Yes	318 (9.3)	52 (16.3)		–	–
		*P* < 0.001			

In the multivariate analysis (Table 5), the prevalence of ever smoking increased with age (*P* = 0.001 for linear trend), was lower in girls (odds ratio [OR] 0.45; 95% confidence interval [CI]: 0.34 to 0.60), differed across religious groups (*P* = 0.004) although not with ethnicity and was related to each of father (OR 1.95; 95% CI: 1.21 to 3.15), mother (OR 3.90: 95% CI: 1.63 to 9.33) and best friend (OR 5.86; 95% CI: 4.31 to 7.96) smoking. The association of Internet access remained significant after adjusted for other factors (OR 1.75; 95% CI: 1.35 to 2.27), although watching football was not significant in the multivariate analysis (*P* = 0.2). More of our respondents had been aware of tobacco adverts or branding on TV than in newspapers or billboards, although exposure in any of these sites was associated with an increased likelihood of having ever tried smoking (Table 4). However, in multivariate analysis, only possessing an item with a cigarette brand logo remained significant. Similar associations were observed with current smoking, although not all reached statistical significance in the multivariate analysis.

**Table 5:** Multivariate analysis of risk factors for cigarette smoking.

	Ever smoking		Current smoking	
	Odds ratio	95% CI	Odds ratio	95% CI
**Age, years**	1.25	1.13 to 1.39	–	–
**Female sex**	0.45	0.34 to 0.60	–	–
**Religion**				
Orthodox Christianity	1.00	–	1.00	–
Protestant	0.59	0.35 to 0.99	0.86	0.31 to 2.43
Catholic	0.79	0.30 to 2.05	2.24	0.37 to 13.59
Muslim	1.71	1.02 to 2.88	1.58	0.73 to 3.41
Other	3.47	1.69 to 7.09	16.89	6.45 to 44.25
**Father smokes?**				
No	1.00	–		
Yes	1.95	1.21 to 3.15		
Not applicable	0.86	0.47 to 1.60	–	–
**Mother smokes?**				
No	1.00	–	1.00	–
Yes	3.90	1.63 to 9.33	11.78	3.28 to 42.23
Not applicable	2.49	1.34 to 4.61	2.89	1.41 to 5.93
**Best friend smokes?**				
No	1.00	–	1.00	–
Yes	5.86	4.31 to 7.96	30.81	14.98 to 63.39
Do not know	1.04	0.78 to 1.39	0.83	0.18 to 3.81
**Access to the Internet at home**				
No	1.00	1.35 to 2.27	–	–
Yes	1.75			
**Possess item with cigarette brand**				
No	1.00	1.78 to 3.25	1	2.25 to 6.27
Yes	2.41		3.75	

CI, confidence interval.

### Alcohol use

One thousand one hundred and sixteen (28.6%) had ever drank any alcoholic product (68 missing) (see Table 3). When asked what they drank, the most common drinks were beer, tella (a traditional Ethiopia beer) and wine. In terms of current drinking, 551 (14.1%) responded positively that they were regularly drinking alcoholic drinks. Of these, 357 were drinking beer and 189 were drinking wine regularly. Those drinking beer did so a median of two (range 0–30) times per week. Those drinking wine did so a median of 1 (range 0–8) time per week. Only 105 replied for how many drinks in total they had in a week; among these respondents, the median was 2 and range 0–30.

### Risk factors for alcohol use

In univariate analysis, age, sex, type of school, grade, religion, ethnicity, father and mother drinking and proportion of friends who drink were significant predictors of ever drinking. Ever drinking was more common in boys and tended to increase with age and grade. Ever trying alcohol was higher in the Tigre and Wolayta ethnic groups and in Christian compared to Muslim groups. Ever drinking was more likely in those with parents who drink and in those with more than 10% of their friends drinking.

In the multivariate analysis (Table 6), ever drinking increased with age (<0.001), was lower in girls (OR 0.48; 95% CI: 0.38 to 0.61), differed across religious groups (*P* < 0.001) and ethnic groups (*P* < 0.001) and was positively associated with both fathers (OR 1.85: 95% CI: 1.49 to 2.29) and mothers (OR 1.72; 95% CI: 1.35 to 2.20) drinking. Alcohol use ever was also positively related to how much students watched football (OR 2.48; 95% CI: 1.87 to 3.29) for watching daily compared to those who never watched it or did not like it. Alcohol use ever was also increased in those with Internet access at home (OR 1.70; 95% CI: 1.52 to 1.89). Similar associations were seen for current drinking.

**Table 6:** Multivariate analysis of risk factors for alcohol use.

	Ever drinking		Current drinking	
	Odds ratio	95% CI	Odds ratio	95% CI
**Age, years**	1.44	1.29 to 1.61	1.31	1.17 to 1.47
**Female sex**	0.48	0.38 to 0.61	0.57	0.43 to 0.74
**Religion**				
Orthodox Christianity	1.00	–	1.00	–
Protestant	0.64	0.46 to 0.90	0.90	0.62 to 1.30
Catholic	1.19	0.54 to 2.64	2.36	1.21 to 4.59
Muslim	0.23	0.15 to 0.35	0.27	0.15 to 0.49
Other	0.47	0.24 to 0.93	0.60	0.15 to 2.33
**Ethnic group**				
Oromo	1.00	–	1.00	–
Amhara	0.99	0.77 to 1.27	0.91	0.73 to 1.13
Gurage	0.74	0.52 to 1.05	0.49	0.27 to 0.88
Tigre	1.49	1.10 to 2.01	1.11	0.79 to 1.56
Wolayta	1.32	0.60 to 2.89	1.16	0.33 to 4.08
Silite	1.36	0.66 to 2.77	2.34	1.12 to 4.89
Other	1.49	0.72 to 3.10	0.62	0.30 to 1.29
**Father drinks?**				
No	1.00	–	1.00	–
Yes	1.85	1.49 to 2.29	1.45	1.05 to 2.01
Not applicable	1.29	0.84 to 1.99	1.34	0.80 to 2.26
**Mother drinks?**				
No	1.00	–	1.00	–
Yes	1.72	1.35 to 2.20	2.00	1.44 to 2.77
Not applicable	1.07	0.72 to 1.60	1.27	0.75 to 2.14
**Type of school**				
Government	–	–	1	–
Private			0.47	0.27 to 0.82
**Access to the Internet at home**				
No	1.00	–	1	–
Yes	1.70	1.52 to 1.89	1.53	1.24 to 1.90
**Football**				
Do not like football/never	1.00	–	1.00	–
Watch it once a month	1.02	0.74 to 1.41	1.00	0.63 to 1.59
Watch it weekly	1.64	1.27 to 2.11	1.73	1.25 to 2.40
Watch it daily	2.48	1.87 to 3.29	2.44	1.58 to 3.78

CI, confidence interval.

### The relation between smoking and alcohol consumption

There was a strong relation between ever having smoked and ever having used alcohol, with 72% of those who had ever tried smoking having ever tried alcohol, compared to only 25% in those who had not tried smoking. In those who had ever used alcohol, 21% had ever smoked, compared to 3% in those who had not used alcohol (*P* < 0.001).

In a sensitivity analysis including all those with missing data on age, the results for prevalence of smoking and alcohol behaviours were marginally higher, but the effects of potential risk factors were very similar to the main analysis.

## Discussion

These data represent the first description of the consumption of both alcohol and tobacco products among adolescents attending Ethiopian high schools that consider exposure to novel risk factors such as televised football in which alcohol advertising is common. Smoking remained relatively uncommon in this population with only 3% reporting current smoking, whereas alcohol use is far more common with 1 in 7 young people drinking alcohol regularly. Smoking and alcohol consumption were both associated with parental smoking and alcohol use as well as with that of their peers. Religion was an important determinant of both behaviours but with different patterns across religious affiliations. Both behaviours were more common in those with Internet access, while daily watching of televised football was associated with over a twofold increase in the risk of currently consuming alcohol.

### Strengths and limitations of this analysis

As developing countries experience both economic growth and technological change, it is becoming important to understand the processes that lead to adoption of unhealthy lifestyles to inform public health interventions. The strengths of these data include the relatively broad range of Ethiopian society that was sampled, including those from both urban and rural schools, as well as the addition of questions on the degree of adoption of a modern lifestyle such as use of mobile phones, the Internet and watching football. Our data also have some limitations however. The cross-sectional study design does not allow causality to be inferred. The response rate was good at 94.1%, but we cannot exclude the possibility of responder bias modifying the associations observed. The period of data collection coincided with a turbulent period in Ethiopian politics that ended in a state of emergency which made data collection challenging. Owing to logistical constraints, we only sampled schools that were accessible from Addis Ababa. As a consequence, the generalisability of these data to other regions in Ethiopia is also uncertain, given the very strong regional heterogeneity in cultures. However, our finding that the use of alcohol and tobacco products tended to occur in the same individuals is consistent with earlier findings in Ethiopia,^11^, ^12^ suggesting that our results are likely to be generalisable. Finally, the low prevalence of smoking may have limited the power to the statistical analysis to determine risk factors.

### Tobacco consumption

The prevalences of ever smoking and current smoking in our study were low in relation to most affluent societies but were similar to other studies in Ethiopia which have given estimates for current smoking of 2.9% in adolescents living in Addis Ababa in 2003 and 4.2% for daily cigarette smoking in school adolescents who lived in Harar town in eastern Ethiopia.^6^ Our finding that males were more likely to smoke than females is consistent with the other studies from Ethiopia of adolescents' smoking habits,^6^, ^13^ with these differences persisting into adulthood.^11^, ^14^, ^15^, ^16^, ^17^ Parental smoking has previously been reported as a risk factor for smoking tobacco in Ethiopian young people^13^ and can be expected to both normalise smoking behaviour at home, which is becoming relatively stigmatised in some societies, and increase availability of tobacco products to young people. Smoking is well recognised to spread through friendship groups, and two other Ethiopian studies in this age group have also reported that having friends who smoke is a strong risk factor for smoking,^6^, ^13^ which is also observed in university students.^14^ This supports the hypothesis that tobacco smoking is a contagious habit that is enabled by social norms that are established by family and peer groups.^18^

Our finding that participants with home Internet access were more likely ever to have smoked is consistent with the observation that Internet media may promote cigarette consumption by making cigarette imagery readily available in a relatively unregulated manner.^19^ Alternatively, there may be confounding by affluence, as those who can afford home Internet may also have more disposable income to purchase cigarettes. We also identified that possessing an item with cigarette branding was a risk factor for smoking. This form of marketing is also called brand stretching^20^ and helps promote specific tobacco brands in a form of subliminal advertising. We were unable to collect data on what the branded items were, but this is an area that deserves more research in Ethiopia and other comparable countries. Only a very small proportion of the females in our study sample smoked, but as this represents a big future market, future studies should consider how media may be used to attract new cigarette users.

### Alcohol use

Our data demonstrated that 29% of the study population had ever tried alcohol and that 14% were regular current alcohol drinkers. These estimates are similar to those from other studies in Ethiopia. A similar survey of high school students living in Addis Ababa in 2010 reported prevalences of 46% for ever drinking alcohol and 26% for drinking alcohol in the past month,^21^ while prevalences from a study of high school students living in eastern Ethiopia were 22% and 10%, respectively.^22^ A large national survey of 20,434 individuals aged 15–24 years living in Ethiopia in 2001 and 2002 gave a prevalence of drinking alcohol at a frequency of at least weekly of 21%,^23^ while a survey of students at Haramaya University in 2013 reported that 20% had drank alcohol in the previous 30 days.^24^ In our data and some^14^, ^22^, ^24^ but not all^21^ comparable studies from Ethiopia, prevalence of regular alcohol consumption was higher in males.

We have identified several relatively novel risk factors for being a current alcohol drinker. The association with parental drinking is consistent with the observation that high school students living in eastern Ethiopia are more likely to drink alcohol if they live with people who use alcohol.^22^ Our observation that living in a rural area is a risk factor for current drinking is consistent with the observation of a high prevalence of hazardous alcohol use for people living in a rural Ethiopian district, with men being particularly vulnerable,^25^, ^26^ although this is also a concern in urban Ethiopia.^27^ Home Internet access was also an independent risk factor for current drinking, possibly because adolescents report greater exposure to alcohol advertising and promotional content than adults via the Internet.^28^

It is well recognised that football is a sport that has received much support from the alcohol industry; an analysis of televised top-class English football from the 2011/12 season reported that references to alcohol products were common at an average of almost two per minute.^9^ As these were generally visual references to alcohol, the potential audience of these images is large as the English Premier League is a brand with a global audience. Cohort studies have demonstrated that there is an association between exposure to alcohol advertising or promotional activity and subsequent alcohol consumption in young people,^10^ and hence, these images from football grounds around the UK are likely to be having a positive effect on alcohol consumption globally. Our findings are consistent with this hypothesis.

### Implications

Tobacco and alcohol use are likely to cause significant harm to health in low- and middle-income countries in the coming decades and indicates that the detrimental health impacts of using these substances will be particularly severe in this group. While there are likely to be many component causes driving increased consumption, for alcohol consumption in young Ethiopians, this study identifies a potential major role for advertising associated with televised football. Future observational studies will allow further understanding of how tobacco and alcohol consumption is adopted in societies where baseline prevalence is low and if appropriate target interventions to the sectors of society that are most at risk.

## Author statements

### Ethical approval

The study protocol was reviewed and approved by the Research and Ethics Committees of the School of Public Health at the Addis Ababa University, Ethiopia, and at the University of Nottingham, UK. Students older than 18 years gave oral consent, while for those between 13 and 17 years, consent was provided by the school masters as consistent with the ethics committee approved protocol. All responsible people in the schools were informed about this study. Participation in the study was voluntary, and verbal informed consent was obtained from each student before data collection. Students were informed that questionnaires were anonymous and confidential. Appropriate measures were also taken to ensure confidentiality of information both during and after data collection.

### Funding

Association of Physicians of UK and Ireland. Tobacco Control Capacity Programme (MR/P027946/1) supported by 10.13039/100014013UK Research and Innovation (UKRI) with funding from the Global Challenges Research Fund.

### Competing interests

None declared.
